# The Urgent Need to Develop Novel Strategies for the Diagnosis and Treatment of Snakebites

**DOI:** 10.3390/toxins11060363

**Published:** 2019-06-20

**Authors:** Harry F. Williams, Harry J. Layfield, Thomas Vallance, Ketan Patel, Andrew B. Bicknell, Steven A. Trim, Sakthivel Vaiyapuri

**Affiliations:** 1 School of Pharmacy, University of Reading, Reading RG6 6AH, UK; h.f.williams@pgr.reading.ac.uk (H.F.W.); harrylayfield@gmail.com (H.J.L.); t.m.vallance@pgr.reading.ac.uk (T.V.); 2 School of Biological Sciences, University of Reading, Reading RG6 6AH, UK; ketan.patel@reading.ac.uk (K.P.); a.b.bicknell@reading.ac.uk (A.B.B.); 3 Venomtech Ltd., Discovery Park, Sandwich, Kent CT13 9ND, UK; s.trim@venomtech.co.uk

**Keywords:** snakebite envenoming (SBE), venom, diagnostics, therapeutics, toxin neutralisation, neglected tropical disease

## Abstract

Snakebite envenoming (SBE) is a priority neglected tropical disease, which kills in excess of 100,000 people per year. Additionally, many millions of survivors also suffer through disabilities and long-term health consequences. The only treatment for SBE, antivenom, has a number of major associated problems, not least, adverse reactions and limited availability. This emphasises the necessity for urgent improvements to the management of this disease. Administration of antivenom is too frequently based on symptomatology, which results in wasting crucial time. The majority of SBE-affected regions rely on broad-spectrum polyvalent antivenoms that have a low content of case-specific efficacious immunoglobulins. Research into small molecular therapeutics such as varespladib/methyl-varespladib (PLA_2_ inhibitors) and batimastat/marimastat (metalloprotease inhibitors) suggest that such adjunctive treatments could be hugely beneficial to victims. Progress into toxin-specific monoclonal antibodies as well as alternative binding scaffolds such as aptamers hold much promise for future treatment strategies. SBE is not implicit during snakebite, due to venom metering. Thus, the delay between bite and symptom presentation is critical and when symptoms appear it may often already be too late to effectively treat SBE. The development of reliable diagnostical tools could therefore initiate a paradigm shift in the treatment of SBE. While the complete eradication of SBE is an impossibility, mitigation is in the pipeline, with new treatments and diagnostics rapidly emerging. Here we critically review the urgent necessity for the development of diagnostic tools and improved therapeutics to mitigate the deaths and disabilities caused by SBE.

## 1. Introduction

Snakebite envenomation (SBE) is a life threatening and traumatising affliction that is unequivocally associated with the world’s most impoverished people [1]. Mortalities from SBE are concentrated in the rural tropics where snakes are in abundance and the agricultural work force is poorly protected. The limited recognition of the scale of the crisis by health authorities around the globe afforded SBE a place on the World Health Organisation’s list of neglected tropical diseases in 2009 (NTD). This was followed by a contentious removal before again being reinstated in 2017 and quickly being made a priority NTD [2,3]. The confusion surrounding SBE as an NTD is somewhat justified: SBE is not limited to the tropics and all other NTDs are caused by pathogens entering the body: protozoa, helminths, bacteria and viruses [4]. Thus, the causative agents are easier to identify and study by comparison to the diversity of pathologies associated with SBE. Indeed, Australia classes snakebite as a non-intentional injury rather than a disease, but with an average of two deaths a year, it is unlike the crisis seen in more impoverished countries [5]. The extent of SBE taking place every year is estimated to be between 1.8–2.7 million [6]. The actual deaths from SBE are purported to be between 81,000–137,000 [7] and nearly 50,000 of these deaths are estimated to take place in India alone [8]. There are a further 8000 in Pakistan and 6000 in Bangladesh [9], while in the Americas despite 60,000 snakebites taking place annually, deaths are estimated to only be in the hundreds [10]. While shocking, the deaths frequently hide a potentially greater issue, which is the disability and consequential loss to the economic workforce. Delays in seeking medical assistance are common, and postponements for just a couple of days can lead to gangrene, compartmental syndrome and amputation [11]. Surviving SBE can also have mental health implications, with survivors seeing a three-fold increase in depressive disorders compared to the general population [12]. Post-traumatic stress disorder also occurred in a further 20% of SBE victims surveyed in Sri Lanka [12]. In West Africa, the disability-adjusted life years (years lost due to disability or early death) from SBE are estimated to be over 300,000 [13]. These figures are ever increasing, due to past data suffering from flaws from under-reporting and victims avoiding hospitals for cheaper and more convenient traditional herbalists.

This staggering epidemiology is unsurprising when compared to more typical diseases. The marked difference between SBE and many of the other NTDs is the diversity involved in the range of associated toxins seen globally. Cholera, for example, (not limited to the tropics and therefore sometimes ignored as an NTD), like SBE causes many thousands of deaths every year (Table 1). Cholera has such a dramatic effect on its victims primarily through one toxin (cholera toxin/CT) released by strains of the *Vibrio cholerae* bacteria. CT triggers a cascade of events which culminate in an influx of salts and water into the intestine, causing the diarrhoea that aids in transmission of the disease to others, and leaves victims to die by dehydration [14]. The disease is the result of one toxin, from one species of bacteria, with one simple and effective treatment. The nematode infections (shown in Table 1) are all a result of roundworms, which have evolved to inhabit the gastrointestinal tract of humans. Despite resulting from a range of species, the same anti-helminthic drugs can easily treat this disorder [15]. In stark contrast, SBE can be the result of bites from hundreds of different snake species, each possessing a multitude of different toxin profiles and leading to a vast array of different pathologies [16]. Treatment is consequently far more complex than the rehydration required to beat the majority of cholera infections [17], and simple prophylactics required to prevent many of the other NTDs (Table 1) [18]. SBE has proved to be an incredibly complex disease to accurately diagnose and treat appropriately. 

**Figure 1:**
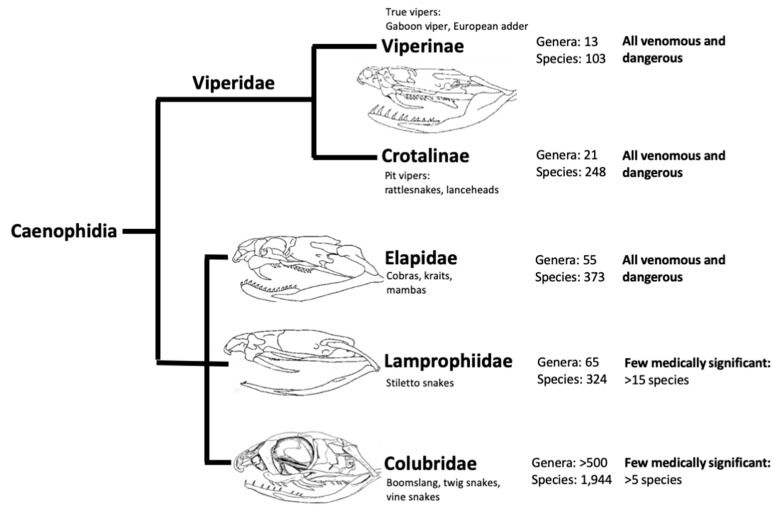
Phylogenetic tree adapted from Reyes-Velasco et al. (2014) [44]. Shows the Caenophidia, a clade including all venomous snakes. The skull diagrams were adapted from published images [45,46]. Number of species and genera were taken from the reptile database [47].

Venoms are essentially cocktails of toxic and non-toxic components: proteins, peptides, metal ions and small organic molecules, including nucleotides, secreted by animals to predate on or defend against other animals. In snakes, venom is a modified form of saliva produced from a pair of venomous glands and delivered by fangs, and found in species from a number of taxa. All venomous reptiles have been grouped in a clade called Toxicofera, within which, another clade, Caenophidia holds all the venomous snakes (Figure 1). The strictly venomous families are Elapidae (elapids) that includes the snakes with fixed front fangs e.g., cobras, kraits, mambas, taipans and sea snakes (sometimes grouped in the subfamily, Hydrophiinae) amongst others; and Viperidae (vipers) which have hinged front fangs allowing longer fangs and deeper tissue penetration. The vipers are further divided into two subfamilies, Viperinae (the true vipers, e.g., Gaboon viper and European adder) and Crotalinae (the pit vipers, e.g., rattlesnakes and lanceheads). Additionally, two other families contain venomous species, although the majority of these families are made up of non-venomous snakes. The first is Colubridae (colubrids), a loose grouping containing over half of described snake species with reported deaths arising from at least five species: the boomslang (*Dispholidus typus*); twig snake (*Thelotornis capensis*); tiger keelback (*Rhabdophis tigrinus*); South American green racer (*Philodryas offersii*) and Peruvian slender snake (*Tachymenis peruviana*) [29], though many others have potentially occurred. The second mixed family is Lamprophiidae (lamprophiids), which contains several venomous (e.g., Atractaspidinae) and non-venomous subfamilies, of note is the genus *Atractaspis*: the stiletto snakes or burrowing asps [16,30,31]. Venomous bites from *Atractaspis* occur across most of sub-Saharan Africa (and some western Asian counties) and occasionally cause fatalities [32] due in part to a lack of specific antivenom over most of the genus’ range. The majority of lethal bites are, however, almost exclusively from members of Viperidae and Elapidae families [33]. The impact of these two families can be oversimplified as largely neurotoxic in the case of elapid bites and haemotoxic in the case of viper bites. Flaccid paralysis and respiratory failure often result from elapid bites. Hypovolemic shock (the loss of >20% blood) leading to heart failure [34,35] alongside acute kidney injury [36] are potential causes of death in viper bites. However, there are some vipers that rely on neurotoxic components, such as the atypical South American rattlesnake (*Crotalus durissus terrificus*) the venom of which contains crotoxin, and Russell’s viper, *Daboia russelii*, which contains U1-viperitoxin-Dr1a [37]. Both of these viperid neurotoxins are pre-synaptically active neurotoxic phospholipase A_2_ (PLA_2_) [38] which are generally seen more commonly in elapid venoms (other neurotoxic viper venoms are known [39,40,41]). Elapids are also not without their exceptions to the rule, in particular the Australian elapids for which the cause of death is frequently cardiac arrest, and coagulopathy is also common due to the high proportion of prothrombin activators in their venoms [5,42,43]. 

## 2. The Complexity of Snake Venoms

**Figure 2:**
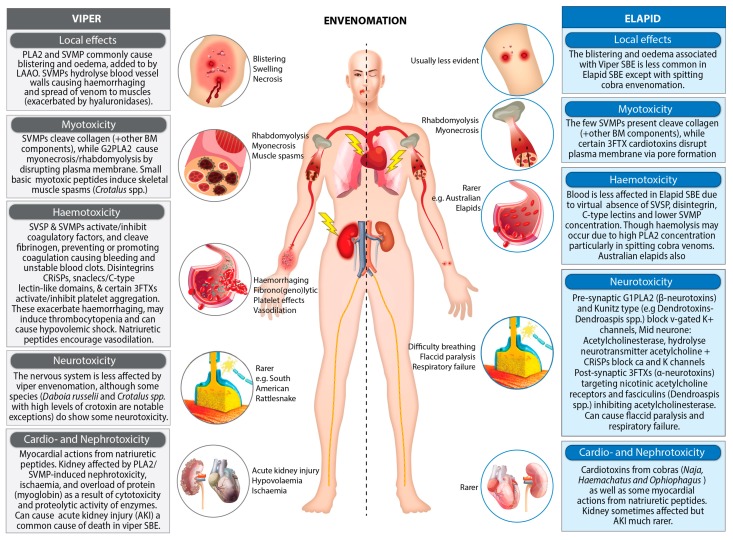
Generalised effects of viper and elapid snakebite envenomation and toxins causing these effects. Inspired by Gutiérrez et al. (2017) [16]. Abbreviations: PLA_2_—Phospholipase A_2_, SVMP—Snake venom metalloprotease, G2PLA_2_—Group 2 PLA_2_, SVSP—Snake venom serine protease, CRiSPs—Cysteine rich secretory proteins, Snaclecs—Snake c-type lectins, 3FTXs—Three finger toxins, SBE—snakebite envenoming, BM—basement membrane.

One of the major difficulties in treating snakebites is the hugely diverse geographic and taxonomic nature of venomous snakes and the consequential variability of venoms [48,49,50]. Many of the 680 or so venomous species of snakes are further split into subspecies each with added levels of diversity in venom compositions to their congeners [51]. As well as this, many undiscovered cryptic species may also exist providing yet further diversity of venom and undiscovered venom components [52]. The variation in venom between these subspecies leads to differences in symptomatology [53] as well as varying levels of antivenom efficacy [54]. Therefore, a thorough knowledge of serpentine systematics is crucial for effective treatment of snakebites [55]. Despite their differences, snake venoms do have many similarities. They are all complex mixtures of hydrolytic enzymes, biologically active non-enzymatic proteins and peptides—these are responsible for the spectrum of their toxic effects (Figure 2) [16]. 

A large number of protein families exist within snake venoms: there are four dominant protein families (phospholipase A_2_, metalloproteases, serine proteases, and three-finger toxins), and six secondary protein families (Cysteine-rich secretory proteins, L-amino acid oxidases, kunitz peptides, C-type lectins, disintegrins and natriuretic peptides) as well as over 36 rarer protein families [56]. These dominant and secondary families form the bulk of snake venoms and are largely to blame for the incredibly broad symptomatology and pathology associated with SBE (Table 2).

### 2.1. Enzymatic Components

*PLA_2_* are a group of esterolytic enzymes present in snake venoms that typically catalyse the breakdown of glycerophospholipids, the main component of biological membranes, into lysophospholipids and a fatty acid (which may be involved with the oxidisation of haemoglobin [57]). However, within snake venoms, many members of this group have lost most of their enzymatic activity and instead bind to various receptors. The snake venom PLA_2_s are split into two groups, group I PLA_2_s are found predominantly in elapid and some colubrid snakes, while group II are found only within Viperidae. Group I are generally β-neurotoxins which act pre-synaptically, sometimes binding to voltage gated potassium channels [58], although multiple mechanisms exist [59,60]. After binding, neurotoxic PLA_2_s can sometimes hydrolyze nerve terminal phospholipids causing permanent neurotoxicity [61]. This has the effect of causing paralysis, while group II PLA_2_s tend to act cytotoxically, predominantly as myotoxins, causing myonecrosis via the disruption of the plasma membrane [62]. Sometimes after hydrolysing membrane phospholipids, non-enzymatic PLA_2_ homologues cause damage to the sarcolemma via hydrophobic interactions [63]. PLA_2_s have further diverse pharmacological functions, however, haemotoxicity [64], postsynaptic neurotoxicity as well as the inhibition and activation of platelet aggregation, cardiotoxicity and anticoagulant effects have also been reported [65,66]. 

*Snake venom metalloproteases* (*SVMPs*) are the most abundant venom enzymes in vipers (also present to a lesser extent in elapids), and include both coagulants (e.g., activation of prothrombin or factor X), and anticoagulants (comprising of integrin shedding and fibrinolytic enzymes [67]). Importantly, they also frequently induce haemorrhaging due to hydrolysis of the endothelial cell basement membrane components around blood capillaries [68]. These also affect muscle fibres impairing their regeneration [69]. These enzymes are in themselves a highly diverse family, and are separated into four groups depending on the domains present: P-I/Group I comprise just a metalloprotease domain, present in all groups. In the venom gland it exists as a zymogen with a pro-peptide domain that is cleaved before activation; P-II/Group II has an additional disintegrin domain, which have been found to be liberated as free disintegrins after processing in some venoms [70]; P-III/Group III has additional disintegrin-like and Cysteine rich domains and P-IV as P-III but with two C-type lectin-like domains attached via disulphide bonds [52,71]. These additional domains afford SVMPs a wide variety of different functions. For example, the disintegrin domains bind integrins blocking their functions in platelets and endothelial cells [72] and have the potential to bind the integrins in muscle cells, colocalising and exacerbating myotoxic effects [69]. Cysteine-rich domains have also been found to inhibit collagen induced platelet aggregation as well as to play a key role in the onset of inflammation [73]. Finally, C-type lectin-like domains, which amongst other functions of SVMPs, are involved in the activation of platelets by the clustering of tyrosine kinase dependent receptors [74].

*Snake venom serine proteases (SVSPs)* mainly affect the haemostasis of victims by proteolytically degrading the blood components (e.g., fibrinogen) as well as modulating various coagulatory factors (e.g., factor V and plasminogen) [75]. Despite the variety of processes SVSPs can affect, the primary function of the majority of studied SVSPs is to cleave fibrinogen, promoting coagulation, but they can also prevent coagulation through dysfibrinogenemia. These are called ‘thrombin-like’ enzymes due to their mimicking of thrombin’s primary function, although SVSPs rarely activate factor XIII which thrombin does in order to cross-link the soluble fibrin clot into an insoluble clot [76]. There are additional SVSPs described as ‘kallikrein-like’ (bradykinin releasing and blood vessel dilating) [77,78], factor V activators (consequently prothrombin activating) [79] and platelet aggregators (via cleavage of protease activated receptors PAR1 & PAR4 [80]) that cause alterations in blood pressure or cause blood to clot [81]. Anticoagulant SVSPs also exist with some found to activate protein C, a proenzyme involved in negatively regulating the coagulation cascade via inactivation of factors V and VIII [82,83], and degrading blood clots by conversion of plasminogen to plasmin. 

*L-amino acid oxidases (LAAOs)* are not an abundant enzyme family, they are, however, found fairly ubiquitously in both elapid and viper venoms [56]. They are glycoproteinaceous flavoenzymes and catalyse the oxidative deamination of L-amino acids. This produces an α-keto acid, ammonia and hydrogen peroxide, all of which can have cytotoxic effects. The hydrogen peroxide produced may additionally lead to the oxidation of haemoglobin seen as a result of some viper venoms [84,85]. They may also induce oedema [86] and apoptosis [87], as well as acting as anti-coagulants via the inhibition of factor IX [88]. These enzymes are, however, still poorly understood, and thought to play some roles in the stabilisation of venom components within the gland or ducts [89] or aid in digestion.

Other enzymes found in much lower quantities in venoms include; acetylcholinesterase, a serine hydrolase which functions synaptically, hydrolysing the neurotransmitter acetylcholine [90]; and hyaluronidases which are known as the spreading factors [91] due to their facilitation of the diffusion of other toxins across the body tissues as well as causing oedema via hydrolysing the hyaluronic acid barrier in the interstitial space [92]. The remaining groups of venom enzymes are thought to be involved more in digestion rather than the immobilisation of prey, and are consequently considered non-toxic by many researchers [93]. However, ignoring the hidden functions of these “non-toxic” components could be imprudent, for example, the ability of nucleases to liberate purines (adenosine in particular) which can act as multifunctional toxins [94]. 

### 2.2. Non-Enzymatic Components

As well as enzymes, there are also a whole host of non-enzymatic venom components, which carry out a variety of different functions. *Three-finger toxins* are characterised by a three-finger fold made up of three loops which protrude from a hydrophobic core [95]. They are found predominantly in elapid venoms, some viper venoms (only via transcriptomics [96]) and also certain colubrid venoms [97,98]. Despite their common structure, they bind to many different receptors and elicit a variety of biological effects [99]. They are typically neuro- or cytotoxic-. The α-neurotoxins, one important group of three finger toxins, bind post-synaptically, to nicotinic acetylcholine receptors found in the skeletal muscle of vertebrates [98], blocking neuromuscular transmission, causing flaccid paralysis and respiratory failure in some cases [100]. Three-finger toxins also include κ-bungarotoxins and haditoxin which operate similarly to the α-neurotoxins [99]; as well as acetylcholinesterase inhibitors—the *fasciculins* of the *Dendroaspis* genus [101]; cytolytic, ion pore forming cardiotoxins (cytolysins) found in cobra venoms [102] and L-type calcium channel blockers and platelet aggregation inhibiting three-finger toxins as well [99]. 

*Cysteine-rich secretory proteins (CRISPs)* are single chain polypeptides widely distributed within venoms, and have been found in the venoms of all the three main families of venomous snakes [68] as well as in some lizard venoms [103]. Like three-finger toxins, CRISPs have a scaffold that is highly conserved and is stabilised via disulphide linkages and exert a wide range of pharmacological activities. Helothermine, a CRISP isolated from the venom of the lizard, *Heloderma horridum horridum* has been found to block calcium [104] and potassium [105] currents in neurons and to lower body temperature in mice [106]. CRISPs have also been documented to inhibit smooth muscle contraction via the blocking of Ca^2+^ channels [107,108] and to block cyclic nucleotide gated ion channels which are significant in many modes of sensory transduction [106].

*Kunitz-type proteinase inhibitors* are small proteins that are found in a range of viper and elapid venoms [109]. While some act to inhibit serine proteases, others have been found to block a large range of ion channels despite high homology. One notable neurotoxic group of Kunitz peptides are called the dendrotoxins, and form the largest component of mamba (*Dendroaspis* spp.) venoms before α-neurotoxins [110]. These proteins have no protease activity and instead interact with voltage gated potassium channels [110,111]. This potentiates the effect of acetylcholine, facilitating its release at the presynaptic nerve terminal causing excitation resulting in involuntary muscle contractions [112]. Synergism between components within venoms is well known [110,113], and some PLA_2_s are even known to act as heterodimers with Kunitz peptides potentiating their combined effects as in β-bungarotoxin from *Bungarus multicinctus* [114] and MitTX from *Micrurus tener tener* [115].

In mammalian systems, C-type lectins typically bind to calcium and sugar residues. However, in snake venoms they are known as *Snake C-type lectin-like proteins* or *snaclecs* and they rarely have the binding loop responsible for this mammalian function but instead bind to a variety of receptors on platelets [74], as well as coagulation factors IX/X [116] and endothelial cells [117]. They have been reported to both inhibit [118] and activate [119] via a number of receptors on platelets including α_2_β_1_, GPIb, GPVI and CLEC-2 [89,120], sometimes causing thrombocytopenia as a result [16,74]. 

The *disintegrins* are a family of polypeptides present in viper venoms, some of which are released from SVMPs while others have independent genes. The majority of disintegrins rely on an RGD (Arg-Gly-Asp) motif, (a tripeptide recognised and used by integrins in cell membrane binding) to inhibit integrin function. Disintegrins are not to be confused with the disintegrin-like domains within certain metalloproteases which instead rely on an ECD (Glu-Cys-Asp) motif [72]. They inhibit collagen induced platelet activation via integrin α_2_β_1_ [121] and can competitively inhibit the binding of collagen to the α1 domain of α_1_β_1_ [122] along with targets on a wide range of other disintegrins [123]. They are predominantly potent inhibitors of platelet aggregation [67], acting primarily upon integrin αIIbβ3, the fibrinogen receptor. Others that do not inhibit platelet aggregation have also been characterised [124]. 

*Natriuretic peptides* have been found in both elapid and viper venoms, although they are found in much higher abundance in viper venoms, occasionally making up as much as 30% of venoms such as within the bushmasters; *Lachesis* genus [125]. These peptides promote natriuresis, that is to say the excretion of sodium into urine by the kidneys, which affects inotropic (speed and force of contractions) and lusitropic (rate of relaxation) myocardial actions, as well as promoting vasodilation causing hypotension [126,127].

There are many other non-enzymatic venom components which have been described as minor protein families [56] these include bradykinin-potentiating peptides (BPPs) which both inhibit angiotensin converting enzyme as well as cleaving bradykinin giving potent hypotensive effects [128]. The presence of growth factors including nerve growth factor (NGF) and endothelial growth factor (EGF) in venom is poorly understood but may be involved in prey incapacitation, with NGFs purportedly causing mast cells to release a mass of chemical mediators and increasing vascular permeability aiding the dispersal of other venom toxins [129]. There are an additional 40 or more rare and unique protein families [56]. These rare families frequently exert only mild, if any, toxic effects such as the *lipocalins* whose function is currently unknown [130]. Others of these families have extremely limited taxonomic distribution within snake venoms such as the *sarafotoxins*, which are a toxic form of the vasoconstrictive endothelins and are only found in the *Atractaspis* genus [95,131]. Small basic myotoxic peptides (also referred to as defensins [56]) which are found in a limited number of *Crotalus* spp. induce muscle spasms and necrosis [16,30] and other proteins such as waglerin which is a neurotoxin found only in *Tropidolaemus* spp. [40]. Venoms are all associated with such different toxin combinations which in some cases work synergistically causing SBE to be an incredibly complex disease to treat. 

Hence, the currently accepted antivenom therapy has numerous drawbacks, and thus, novel strategies are being employed worldwide to improve the treatment of this disease. Real improvements to the treatment will to some extent depend upon better diagnostic methods.

## 3. Antivenom (Anti-Snake Venom/Venin/ASV) and Its Associated Problems

Antivenom is the only effective and accepted treatment for systemic SBE yet to stand up to rigorous scientific testing and has single-handedly saved the lives of those suffering SBE for over a century [132]. 

Despite this, there are a number of major problems associated with antivenom: poor stability in liquid form, adverse reactions, often poor efficacy and great difficulties associated with production, which is frequently too expensive for those most in need. Antivenoms are made via the hyper-immunisation of an animal, typically large mammals e.g., horses and rare instances of manufacturers using sheep and donkeys [133]. The size of these animals means that large volumes of plasma can be collected, allowing larger volumes of antivenom to be generated [134]. This is produced by exposing the animal’s immune system to a single venom leading to the creation of monovalent/monospecific antivenoms, or multiple venoms to produce polyvalent/polyspecific antivenoms. The animal’s immune system responds by raising antibodies (particularly immunoglobulin G [IgG] in mammals) that bind specifically to immunogenic antigens present in the venom/s [135]. The plasma is then separated from the blood by centrifugation or sedimentation procedures and erythrocytes can then be reinfused into the animal [132]. Further purification then occasionally takes place to reduce non-immunoglobulin serum proteins in some antivenoms (CroFab) reducing non-selective effects. Non-specific immunoglobulins are sometimes also removed via affinity chromatography, and digestion by pepsin or papain is sometimes used to remove the Fc regions resulting in F(ab’)_2_ or Fab fragments respectively which are used by the majority of western antivenom producers, though whole IgG is also used [133]. 

### 3.1. Reproducibility Issues Associated with Antivenom Production 

In reality, antivenoms are challenging to produce. Not only do the very same species causing the life-threatening bites have to be milked for their venoms—a high risk task for the personnel involved, but this toxic secretion then has to be injected into an animal at a safe (non-lethal) dose or detoxified in a way so as not to lose immunogenicity. These aforementioned issues cause antivenom generation to be inherently problematic, and can cause stress for the animal, the upkeep of which is already expensive without stress threatening poor immune responses and consequential yields of antivenom [136,137]. The process of production is not only extremely time consuming with low yields, but is also associated with huge batch-to-batch variability [138,139], unsurprising when injecting venoms, which vary greatly, into animals, whose immune systems will have hugely varied responses to the antigen. In order to mitigate these difficulties it is suggested that pooled venoms from at least 20–50 specimens from the same geographical location are used [132]. These can, where available, be compared to national reference venoms for quality control and undergo biochemical characterisation (SDS-PAGE, HPLC, enzymatic activities, etc.) as evidence of consistency. Antivenoms are prepared from pooled plasma/serum and then require rigorous testing to find the median effective dose (ED_50_), i.e., the volume of antivenom required to protect 50% of a population injected with the venom [132].

### 3.2. Relative Instability of Antivenom 

The instability of liquid antivenom reduces its availability in the remote regions of developing countries where it is most needed and lyophilised preparations are problematic. In liquid form, antivenom requires preservatives, as well as, and more problematically, refrigeration at between 4 °C and 6 °C to maintain its potency. Lyophilised or freeze-dried products are also available, but to minimise cost and maximise ease of use, some are distributed in liquid form [140]. Moreover, warmer temperatures can lead to the formation of protein aggregates in liquid antivenom, which increase the chances of adverse reactions [141]. The relative instability results in antivenom being sold with expiry dates and warnings for avoiding the use of antivenom that has undergone multiple freeze-thaw cycles, despite there being some evidence that neither of these have significant effects on antivenom efficacy [142]. Where antivenom shortages have been identified, such as for the North American Coral Snake antivenom, shelf life extension programs (SLEP) have validated stability over that predicted extending their usage period [143]. This prevents the local distribution of antivenom and contributes to over two thirds of snakebite victims preferentially choosing traditional healers over hospital treatment in several parts of the world, where snakebite is a major concern [144].

There have also been some studies suggesting that as well as being less prone to triggering adverse reactions, camelid immunoglobulins may belong to a more thermally stable subclass of IgGs [145] which could help to overcome the need for refrigeration [146]. Despite improved thermostability, this study was not really designed to replicate the variation in temperature that antivenom would undergo over the course of several years in a tropical country.

### 3.3. Adverse Reactions to Antivenom

Antivenom invariably contains immunogenic proteins, which activate the immune system of patients, and causes them to suffer from adverse reactions. These are split into two types: acute (anaphylactoid or pyrogenic) and delayed ‘serum’ sickness (now plasma as most antivenoms are plasma-derived) type reactions [147]. Early reactions may be triggered immediately but can take up to an hour for onset of the symptoms. They are associated with mild symptoms such as urticaria (hives), coughing, vomiting, diarrhoea, headaches and nausea, though severe systemic anaphylaxis can also develop, and is associated with bronchospasm, hypotension and angioedema [148]. Pyrogenic reactions may also occur in response to endotoxins from bacterial contaminants of the antivenom [149]. They are characterised by fever, vasodilation, reduction in blood pressure and shaking chills. Late reactions usually occur several days after the initial dose of antivenom and are a form of type III hypersensitivity, which is caused by a build-up of immune complexes inadequately cleared by the immune system [149]. They give similar symptoms to early reactions, but additional symptoms include joint pain, adenopathy, albuminuria and rare cases of encephalopathy. Anaphylactic reactions are frequently treated successfully using prophylactic drugs such as adrenaline and hydrocortisone [150] which serves to reduce capillary permeability and bronchospasm in people suffering from early adverse reactions [148]. 

The frequency and nature of adverse reactions depends in part on the level of antivenom purification. Crude first generation antivenom has caused adverse reactions in up to 54% of patients [151], but it can be affinity purified to produce second generation antivenoms (pure immunoglobulin mixes without any plasma proteins), consisting of just the whole immunoglobulins (IgG), which some studies have found to decrease adverse reactions to less than 25% of patients [152]. 

By removing the Fc (fragment crystallisable, or tail) regions of these antibodies enzymatically with pepsin or papain, third generation antivenoms are created. The use of papain breaks the hinge regions resulting in Fab fragments (these portions bind the toxin epitopes) [153] while pepsin will initially leave the hinge region intact forming F(ab’)_2_ fragments, although prolonged digestion could result in Fv fragments (fragments containing the variable region) which may only bind to antigens temporarily [154]. The removal of Fc regions is assumed to result in fewer adverse reactions [16], with Fab causing only minor adverse reactions when compared to IgG or F(ab’)_2_ [154]. 

Indeed, despite all the stages of purification, complete neutralisation by antivenom is rarely achieved [155] and a large percentage of IgG found in antivenom may not be therapeutically useful [146]. In addition to the enhanced stability of camelid immunoglobulins mentioned above [156,157], their immunoglobulins lack the light chains that are present in ovine and equine antibodies [158] and have also been found to bind to epitopes not bound by other mammalian IgGs [159]. However, all these developments into antivenom over the years still fail to address two of the treatment’s biggest drawbacks: stability and cost, which together seriously limit the availability of antivenoms to those most in need.

### 3.4. Expense of Antivenom

In addition to animal maintenance, when the level of immunoglobulin purification increases, the price of treatment also increases. Although the more expensive and effective generations of antivenoms are of some use in the western world where they are mostly affordable, in third world countries where snakebites are most prevalent, these extortionate antivenoms can cause victims to be financially as well as physically crippled from a snakebite, the financial burden of which can often extend to their friends and family [160]. In India, treatment can cost up to US$5000, more than double India’s GDP per capita and representative of over ten years salary for a typical farm worker [160]. Likewise in the USA, treatment (including antivenom) costing as much as US$153,000 has been reported [161] though more expensive treatments are likely to have taken place. This does not mean it is impossible to make a cheap antivenom, as an effective antivenom is reported to have been developed from a Nigeria/UK collaboration that is available at just US$40 per treatment, at which price antivenom is considered one of the most cost effective treatments in the world [136]. However, after the expense of clinical trials and hospital charges, treatment in some countries may have cost 1000 times the production cost of one vial of antivenom [161]. 

The majority of snakebites occur in rural settings, these are areas far from the hospitals and where electricity is required for refrigerating and administering antivenom [30]. The instability of antivenom means that keeping supplies in the unrefrigerated but most needed regions is impossible, and even if it were possible, the difficulty of administration combined with high probability of adverse reactions would render it of little use to untrained individuals without adequate tertiary care facilities. The dosage is complex, with hugely different mean effective doses depending on venoms targeted and quantity of venom delivered. Mean effective doses from 47 mL [162] to 180 mL [163] of antivenom have been reported and there is some evidence that larger doses can be less effective [164]. There are frequently drugs co-administered with antivenom such as broad-spectrum antibiotics to treat wound infections around the bite site which may occur due to the oral flora present in the mouths of snakes being introduced into bite victims [165,166], which may also lead to sepsis if untreated. There is also growing evidence that venom glands from many species contain a viable microbiome which may directly contribute to wound infection [167].

Such an expensive and complex medicine will always be difficult to distribute widely enough to give protection to the millions of people living alongside potentially deadly snakes. The dangerously low supplies of antivenom have been described as a ‘crisis’ [168] and available supplies are insufficient in both quantity and quality with limited—if any—preclinical assessment data available [137]. A more practical alternative therapeutic approach would therefore be of great merit, particularly to the developing world. Preventative measures such as wearing rubber boots [169] when harvesting and sleeping under mosquito nets [170] are also important to reduce reliance on therapy alone, though the purchase and distribution to all those in need is unrealistic.

Despite the issues with antivenom, as of yet it is the only medicine proven in the treatment of SBE in humans. The cases where it has been ineffective should not detract from its ability to save people even in the late stages of envenomation [171]. Indeed many of the problems arising from the use of antivenom can be minimised by adhering to the WHO guidelines for the management of snakebite [148]. Guidelines which could also serve to reduce the administration of dangerously large doses of antivenom, with reports of doses of 200 vials or two litres of antivenom in some rare cases [172]. 

## 4. Diagnosis of Snakebites 

For a long time, the diagnosis of snakebites has relied almost entirely on the symptomatology as well as a detailed clinical history of the symptoms, and of the offending snake. For rapid assessment, five brief questions to help with this have been described [16]:

1. Where were you bitten? Leading to examination of bite site.
2. When were you bitten? In recent bites symptoms may be absent.
3. What were you doing when you were bitten? Activity may be diagnostical.
4. Where is the snake that bit you or what did it look like? Actual snake or photo can aid in diagnosis.
5. How are you feeling now? Check for further symptoms of envenoming.

In some situations, notably bites from the *Bungarus* genus, victims may wake paralysed as this genus frequently bites people indoors, at ground level, during the night [173]. In these sorts of situations there is no way of verbally confirming a bite and clinical or laboratory diagnosis have to be employed. Improvements to snakebite diagnostics would not only rule out administration of antivenom in cases of dry bites and bites from species not covered by the antivenom or non-venomous but may also begin to quantify the scale of envenoming and quantity of antivenom required, as well as paving the way to more case-specific treatments.

### 4.1. Clinical Diagnosis 

The clinical symptoms to be seen first in viper envenomation are blistering, swelling, bleeding, necrosis and pain. While the first signs of neurotoxicity from elapid envenomation is ptosis caused by ophthalmoplegia (paralysis of facial and extraocular muscles) which can descend into cyanosis and a decrease in ventilatory capacity in the run up to flaccid paralysis. Further diagnosis relies on the presence of multiple markers, and systemic envenoming can be confirmed by peripheral neutrophil leucocytosis (an increase in the number of neutrophils in response to the venom), or abnormal haematocrit (ratio of red blood cells to blood) which can be an indicator of haemorrhaging (low haematocrit) or haemoconcentration due to plasma leakage from increased permeability of capillaries (high haematocrit) [16]. Incoagulable blood after bites from vipers, certain elapids and colubrids have caused the 20-min whole blood clotting test to be the mainstay of snakebite diagnostics for decades. This involves leaving a small sample of victim’s blood in a glass tube for 20 min and then ascertaining whether it has clotted. This can be a good indicator of consumption coagulopathy and usually the presence of procoagulant [30] proteins in the venom of the offending snake [173,174]. Despite being worthy of acting as a diagnostical tool in some settings [175], it is primitive and there are instances of it providing false information [174] and hence, better diagnostics are undoubtedly required [176].

### 4.2. Venom Detection Kits

The Australian Commonwealth serums laboratory snake venom detection kit (CSL-SVDK) [177] which relies on an enzyme-linked immunoassay procedure is currently the only SBE detection device commercially available. This kit is less of a snake identification device than a tool for matching one of Australia’s five monovalent antivenoms—Tiger snake (*Notechis*), Brown snake (*Pseudonaja*), Black snake (*Pseudechis)*, Death adder (*Acanthophis*) or Taipan (*Oxyuranus*)—to the envenomation. For example, the tiger snake immunotype will also neutralise a range of other species including Lowland Copperhead (*Austrelaps superbus*) as well as some *Pseudechis*, *Tropidechis* and *Hoplocephalus* species [177]. The major problem with snakebite diagnostics is the cross reactivity seen between venoms, as some of the proteins in each venom overlap, detection devices are rarely species specific and will detect a range of species when using immunological techniques. This problem is especially evident with the CSL-SVDK which has been recorded giving false positives with bites from species considered completely non-venomous [178,179]. More specific molecular methods are being developed, but are inappropriate as point of care devices, although potentially beneficial in corroborating reliability in more appropriate point of care devices [180]. The production of lateral flow assays (LFA) which can quickly and qualitatively differentiate between bites allowing more specific antivenoms to be used is necessary. Research is well under way on the use of lateral flow devices to detect snakebites, and a device capable of differentiating between Indian cobra, *Naja naja* and Russel’s Viper, *Daboia russelii,* envenomation in India has been developed [181]. Similarly, in Taiwan an LFA device to detect either haemorrhagic bites (*Trimeresurus stejnegeri* & *Protobothrops mucrosquamatus*) or neurotoxic bites (*Bungarus multicinctus* and *Naja atra*) and discriminating between which of the two bivalent Taiwanese antivenoms to use has also been reported [182], which is progress from the enzyme-linked immunosorbent assay currently in use and single band LFA previously reported [183]. 

### 4.3. Improving the Diagnosis of SBE 

A large percentage of snakebites are considered dry bites; where a venomous species has delivered no venom or bites from non-venomous species. Alternatively, bites from venomous species with a venom that has mild or non-lethal effects on humans, but has instead evolved to defeat amphibian, reptilian, icthian or avian prey can lead to confusion regarding the state of envenomation [184]. These bites may still be presented to hospitals, using antivenoms unnecessarily, taking hospital beds and the time of healthcare professionals. A basic device simply able to differentiate between someone suffering a life-threatening bite or a dry bite would therefore provide confidence for clinicians including the less experienced personnel in rural regions and overcome the problems associated with lack of experience in administration of antivenom. A simple device may enable victims to confirm SBE and seek prompt hospital treatment instead of resorting to traditional healers.

In the case of true envenoming, that is to say the injection of a potentially life-threatening venom, immediate transfer to hospital and administration of the correct antivenom saves countless lives [16]. However, choosing the correct antivenom is frequently not an option, as there is often only one choice. In many of the problem areas the antivenom used is polyvalent; in India an antivenom raised against the ‘Big Four’—Indian Cobra (*Naja naja*), Russell’s Viper (*Daboia russelii*), Indian krait (*Bungarus caeruleus*) and Saw-scaled viper (*Echis carinatus*)—is used [185]. Similarly, the most widely used antivenom in Africa is polyvalent (SAVP) and is raised against 11 species: Black mamba (*Dendroaspis polylepis*), Green mamba (*Dendroaspis angusticeps*), Jameson's mamba (*Dendroaspis jamesoni)*, Cape cobra (*Naja nivea*), Snouted cobra (*Aspidelaps lubricus*), Egyptian cobra (*Naja haje*), Forest cobra (*Naja melanoleuca*), Gaboon viper (*Bitis gabonica*), Mozambique spitting cobra (*Naja mossambica*), Puff adder (*Bitis aerietans*) and Rinkhals (*Haemachatus hemachatus*) [186], meaning a small fraction of the antibodies is specific for each snake bite. With typically just one choice of antivenom, clinicians have the binary choice of whether or not to administer the only antivenom available. A point of care test would prevent unwarranted and wasteful administration of this life-saving medicine, which despite new antivenoms being produced is still in very short supply (~2.5% of projected needs) [187]. It could also enable doctors to become less reliant on presented symptoms, and to be more conclusive in identifying the offending snake, depending on a device’s specificity for different taxa (families, genera, species, etc.). After the development of diagnostical devices, the many emerging possibilities for alternative treatments can be used with confidence and reliable epidemiological data can be gathered, allowing more rational distribution of antivenoms or novel treatments.

## 5. Future Treatment Approaches for SBE

Current animal-derived antivenoms are clearly antiquated, as are the huge number of ineffective herbal remedies still used and sought around the world [188] but the difficulties in evolving from this treatment are endless. That said, around the world a lot of research into future strategies is taking place and they have been reviewed in detail [189,190,191,192] as have the design considerations [193] which should be carefully considered by health care authorities before plunging forwards with unrealistic solutions. The majority can be split into small molecular inhibitors and protein or nucleic acid-based technologies.

### 5.1. Small Molecular Therapeutics (SMTs)

As mentioned previously, a large proportion of venoms (particularly viperidae) are made up of enzymatic components, with specific active sites, that frequently depend on just three amino acids—the catalytic triad. Therefore, compounds that can block this site prevent the enzymatic function of that venom component. A number of different small molecular therapeutics have been causing excitement in recent years. The use of such compounds can be expedited by molecular docking studies and promising results have been obtained in this manner [194]. The foremost SMTs are varespladib and its orally available prodrug methyl-varespladib which are repurposed drugs for treating acute coronary syndrome, which shows potent inhibition of the secreted PLA_2_ found in a range of snake venoms [195]. Although an inhibitor of just one venom enzyme family, it has been shown to improve survival in a large range of experimental envenomations. This has been shown to inhibit the anticoagulant and haemorrhagic aspects [196,197], as well as the myotoxicity caused by Group I and II PLA_2_ [198]. As has been previously noted, however, many snake venoms are devoid of PLA_2_ (notable is the black mamba *Dendroaspis polylepis*, with <0.1% PLA_2_ [110]), and varespladib is unlikely to be efficacious against bites from these species [190]. The second most abundant enzymatic group after PLA_2_ are the SVMPs (see Table 2), for which the matrix metalloprotease inhibitor batimastat and orally available prodrug marimastat have been shown to effectively abrogate the haemorrhagic and necrotic effects of these enzymes [194,199]. Both these SMTs, varespladib and batimastat/marimastat have side-effects however, significantly increasing the incidence of myocardial infarction [200] and inhibiting vascular growth [201], respectively. This causes unnecessary administration to be inadvisable and corroboratory diagnostics to be an important first step prior to administration. Acetylcholinesterase inhibitors such as neostigmine and atropine have also come under investigation with promising results in reducing mortality from some elapid venoms [202,203,204]. Nanoparticles (particles <100 nm) are also under investigation [205], and C60 fullerine (a spheroidal carbon molecule) has shown some antivenom properties in an insect model [206] although of course this is a far cry from the mammalian system involved in human SBE.

### 5.2. Protein, Peptide and Oligomer Based Technologies

In addition to antivenoms, other large biomolecular therapies are being investigated. Monoclonal human single chain variable fragments (scFvs) [207] as well as full monoclonal human IgGs [112] (known to have longer half-lives and different Fc-dependent effector functions) have also both been developed. Fewer adverse reactions and promises of cost competitivity [208] indicate these technologies could be key in the inhibition of various components of snake venoms in the future. This research has highlighted the huge potential of recombinantly expressed oligoclonal mixtures in the neutralisation of venom toxins [112]. The large size of these molecules is a double-edged sword, however, and although their half-lives are prolonged compared to small molecules, their speed of distribution is reduced. Thus, reduced tissue penetration impacts the ability to reach areas affected by the tissue damage and necrosis associated with viper bites [209]. The single domain antibodies (sdAb), specifically those based on heavy chain variable domains, have begun to receive some attention. This research has predominantly focused on the V_H_H fragments from camelids (nanobodies). These nanobodies are small, specific, stable and show high affinity for their epitopes making them a promising lead in potential future antivenoms [210]. The possibility of further improving their thermostability is also interesting [211]. Diabodies which are antibody-based dimers which bind antigens divalently and are composed of two single chain fragments [212], have been shown to neutralise neurotoxins [213] and benefit from retaining the benefits of an IgG molecule—with two binding sites, but being approximately one third of the size. 

The use of aptamers, which are short sequences of DNA or RNA that bind to specific targets also show promise and have been shown to inhibit toxins from cone snails [214] as well as α-bungarotoxin and cardiotoxins [215]. Research is also being carried out on a large range of alternative binding scaffolds (AbScaffs) which due to low cost of production, high stability and engineerability could play a key role in future therapeutics for SBE [216].

A number of ABScaff proteins have been put forward as having potential in venom toxin neutralisation including affimers based on phytocystatins; adnectins (monobodies) based on a fibronectin domain and affibodies based on an Fc-binding staphylococcal domain, amongst many others [216]. Although yet to be put to use as antivenoms, these ABScaffs hold huge potential. They mostly rely on diversifiable loop regions, the insertion of peptide or nucleotide sequences into which allow molecular recognition and flexibility in binding regions. The major problem with these scaffolds arises with their very short half-lives and all suffering from rapid clearance by the kidneys. The *in vivo* half-life of aptamers can be as low as two minutes [217] though PEG-ylation can increase this as can fusion to antibodies [218] which has the potential to increase half-lives and turn these scaffolds into realistic treatments. Such PEG-ylation and fusion to larger molecules defeats their foremost advantage: their small size, and would inevitably have cost implications. 

The Fc domains found on antibodies are markers, allowing these proteins to be recycled back into the blood stream: proteins not possessing this region required for interaction with the neonatal Fc receptor are subjected to catabolism via lysosomal degradation [219,220], therefore, conjugation of promising ABScaffs to Fc domains has the potential to increase the half-lives of lead compounds.

## 6. Diagnostics Feeding into Treatment

Improved diagnostics are essential in not only differentiating between diverse venoms but allowing clinicians to act before symptoms of SBE develop and expediting the use of standard protocols rather than being forced to rely on their own judgement as is too frequently the case [172]. Hence, simple diagnostics would boost the production of more specific treatments. At the animal-derived antivenom level: monovalent, bivalent and genus specific antivenoms could be produced and used with confidence. Family specific detection devices, differentiating between a viper and elapid bite, could allow the two major polyvalent antivenoms (for Africa and India) to be divided, at least by family. The ‘Big Four’ antivenom could become two bivalent antivenoms; with one raised against saw-scaled viper, *E. carinatus*, and Russell’s viper, *D. russelii*, venoms and the other against Indian cobra, *N. naja*, and Indian krait, *B. caeruleus* venoms. Similarly, African antivenoms could potentially be split into an elapid and viper antivenom. This would mean a higher proportion of each vial would be specific to the family-specific envenomation effects suffered by the patient. 

**Figure 3:**
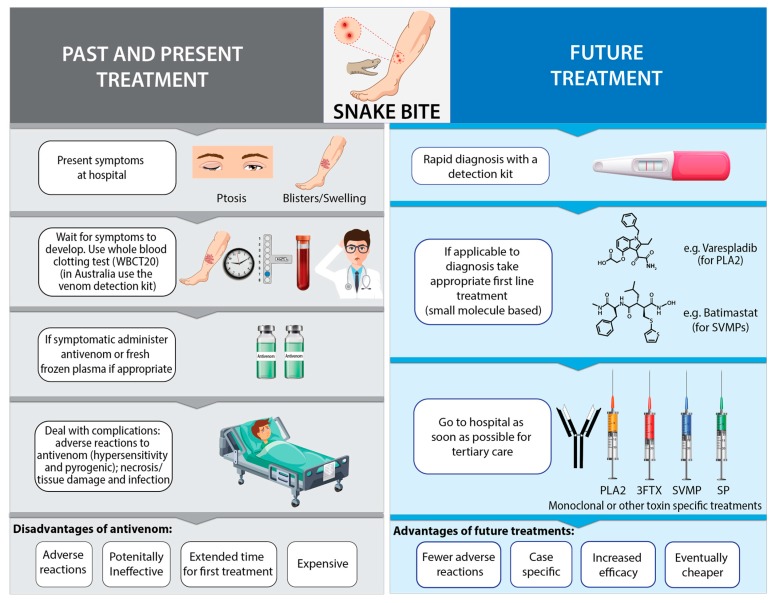
Comparison of current and future events involved in the diagnosis and treatment of snakebite envenoming.

Increasing advancement of kits able to detect specific toxins, could allow the envenoming species to be inferred, allowing treatments to begin targeting toxins present in the blood and those local toxins associated with the bite from a species. The cheap and heat stable SMTs could be made available locally in areas of high risk, allowing pre-hospital adjunctive treatments to be administered after ascertaining envenomation that could lower risk of paralysis and tissue damage [189]. Further surveillance and secondary treatment at hospital may then be sought, with more specific monoclonal, ABScaff or any other improved treatments administered (Figure 3).

## 7. Conclusions

SBE continues to be one of the most neglected tropical diseases, associated with one of the largest annual burdens of all the NTDs (over 1 million people in sub-Saharan Africa alone [221]) and one of the highest mortality rates. We need novel strategies in the diagnosis and treatment of SBE. Although they are required, are they realistic? Improved diagnosis involves fewer hurdles and will have a direct impact on patient outcomes, but the development and licencing of a superior therapy to treat snakebites other than antivenom will take time. The World Health Organisation’s SBE working group has recently been developing a new strategy “WHO snakebite envenoming road map” [222] which aims primarily to augment antivenom production, the only available treatment for snakebite. The stockpiling of antivenoms by the WHO will increase affordability and access to this lifesaving drug worldwide while scientists continue to improve potential future therapies. However, diagnostics are in immediate need of improvement to prevent inappropriate administration of a drug which in much of its range is a precious resource [137]. The strategy appreciates that a focus on new treatments and effective diagnosis also needs to be made and that the acceleration of preclinical and clinical testing of treatments such as Varespladib may well improve hospital survival [222].

With climate change, the overlap between humans and venomous snakes seems likely to increase, as tropical margins also increase [223]. Added to which, poor waste management and the unrelenting rise of rodents alongside the continued expansion of humans into snake territory promises to support the rise of SBE throughout the Anthropocene unless something is done. Understanding the risk factors contributing to SBE is already leading to simple and cheap methods of prevention such as wearing boots and sleeping under mosquito nets. This needs to continue as we need to learn to live alongside the natural world, working with it rather than against it; for example, despite the dangers of electricity, it has become an essential part of our life. Similarly, even though SBE is dangerous, we have to learn to live with snakes to maintain natural biodiversity. While the complete eradication of SBE would require an impossible and unethical worldwide extermination of all venomous snake species, the disease can be mitigated. Reducing predators like this will bring further problems from increased pest species and disease vectors. The SBE working group has a strategy that has the potential to carry out its promise of halving deaths by 2030 [222], by which time a non-animal-derived antivenom might just have been approved. Although with reports of drug development and approval taking over 15 years and costing up to USD$12 billion, this is by no means a foregone conclusion [224]. Novel strategies are undoubtedly emerging and are indeed required if this disease is ever to cease being a frequently deadly medical emergency.

## Figures and Tables

**Table 1:** A comparison of snakebite envenomation (SBE) alongside other traditional major neglected tropical diseases. Sorted based on deaths. Adapted and updated from Hotez et al. (2007) [19].

Disease (Source of Data)	Causal Species	Estimated Deaths/An	Global Prevalence	Population at Risk	Clinical Manifestations	Treatment	Diagnostics
**Snakebite Envenomation** [7]	Snakes: >90 Genera, >700 Species	81,000–137,000	Up to 2,700,000	6–7 Billion	Neurotoxicity and paralysis or cardiovascular toxicity and hypovolemic shock. Cytotoxicity leading to tissue damage and amputation.	Anti-venom	Fang marks, local tissue damage, immunoassay (Aus) Clinical/laboratory markers give other indications
**Cholera** [20,21]	Bacterium: *Vibrio cholerae*	68,400	2,800,000	1.4 Billion	Watery diarrhoea	Oral or intravenous rehydration	Stool examination
**Leishmaniasis** [20]	Protist: *Leishmania* spp. Transmitted by female sandflies; *Phlebotomus/Lutzomyia* spp.	24,200	12,000,000	350 Million	Cutaneous and mucocutaneous disease, kala-azar	Anti-monials, amphotericin B, pentamidine, miltefosine	Biopsy
**Chagas’ Disease** [20]	Protist: *Trypanosoma cruzi*	8000	5,700,000	70 Million	Cardiomyopathy, megacolon, Mega esophagus	Benznidazole, nifurtimox	Blood smear
**Schistosomiasis (Bilharzia)** [20]	Trematodes: *Schistosoma* spp.	4400	207,000,000	779 Million	Hematuria and urogenital disease, intestinal and liver fibrosis, growth and cognitive delays	Praziquantel	Stool examination
**Human African Trypanosomiasis** [20]	Protist: *Trypanosoma brucei* amongst other species. Transmitted by tsetse flies; *Glossina* spp.	3500	300,000	60 Million	Sleeping sickness	Pentamidine, suramin, melarsoprol, eflornithine	Biopsy or blood smear
**Ascariasis** [20]	Nematode: *Ascaris lumbricoides*	2700	807,000,000	4.2 Billion	Malnutrition, growth and cognitive delays	Albendazole/mabendazole	Stool examination
**Trichuriasis** [22,23]	Nematode: *Trichuris trichiura*	Deaths rarely direct	604,000,000	3.2 Billion	Inflammatory bowel disease, growth and cognitive delays	Albendazole/mabendazole	Stool examination
**Hookworm Infection** [22,23]	Nematodes: *Ancylostoma duodenale/Necatora americanus*	Deaths rarely direct	576,000,000	3.2 Billion	Anemia, malnutrition, growth and cognitive delays, poor pregnancy outcome	Albendazole/mabendazole	Stool examination
**Lymphatic Filariasis** [24,25]	Nematodes: *Wuchereria bancrofti, Brugia* spp.	Deaths rarely direct	120,000,000	1.3 Billion	Adenolymphangitis, lymphedema, hydrocele	Ivermectin/diethylcarbamazine (plus albendazole)	LFA test strip (Alere)
**Trachoma** [19]	Bacterium: *Chlamydia trachomatis*	Deaths rarely direct	84,000,000	590 Million	Trachomatous folliculitis and inflammation, trichiasis, blindness	Surgery, aziromycin	Clinal diagnosis using loupes (magnifiers)
**Onchocerciasis** [26]	Nematode: *Onchocerca volvulus.* Transmitted by blackflies; *Simulium* spp.	Deaths rarely direct	37,000,000	90 Million	Onchocerca, skin disease, blindness	Ivermectin	Biopsy/slit lamp examination/antibody tests
**Leprosy** [27]	Bacterium: *Mycobacterium leprae*	Deaths rarely direct	200,000	ND	Lepromatous leprosy, tuberculoid leprosy	Multidrug therapy, rifampicin, clofazimine, dapsone	Biopsy
**Dracunculiasis** [28]	Nematode: *Dracunculus medinensis*	Deaths rarely direct	30	ND	Disfiguring ulcer, secondary bacterial infection	Metronidazole/thiobendazole adjunctive to self-care and stick therapy	Clinical presentation

**Table 2:** The major enzymatic (grey) and non-enzymatic (blue) proteins found in snake venoms and their primary functions. The table was adapted from Warrell (2010) [30] and abundance data were created using data from 132 snake species (42 members of Elapidae, 20 Viperinae and 65 Crotalinae). These data were provided in Tasoulin & Isbister (2017) [56] and data were used with the authors’ permission.

Venom Component	Approximate Abundance (% (±SD))			Major Described Functions
	Elapidae	Viperinae	Crotalinae	
Phospholipase A_2_ (PLA_2_)	31 (±24)	22 (±17)	22 (±20)	Presynaptic neurotoxicity (β-neurotoxins), membrane phospholipolysis, haemolysis, myotoxicity, necrosis and inhibition/activation of platelets
Snake venom metalloprotease (SVMP)	3 (±3)	35 (±20)	36 (±20)	Haemorrhaging, fibrin(ogen)olytic activity, endothelial damage and myotoxicity
Snake venom serine protease (SVSP)	1 (±1)	12 (±9)	16 (±14)	Hypotension, fibrin(ogen)olytic activity and bleeding
L-amino acid oxidase (LAAO)	1 (±2)	2 (±2)	5 (±4)	Apoptosis, oedema, cytotoxicity via products and anticoagulant effects via inhibition factor IX
Three-finger toxin (3FTX)	55 (±27)	NA	NA	Postsynaptic neurotoxicity via binding of cholinergic receptors (α-neurotoxins), cardiotoxicity, myotoxicity and cytotoxicity
Kunitz type serine protease inhibitors (KSPi)	4 (±10)	3 (±6)	NA	Neurotoxicity via binding of voltage gated potassium channels or anticoagulopathic effects due to serine protease inhibition
Cysteine rich secretory protein (CRiSP)	2 (±3)	4 (±4)	2 (±2)	Smooth muscle inhibition via blocking of calcium channels
Natriuretic peptides	1 (±1)	1 (±3)	7 (±9)	Promote excretion of sodium by kidneys causing hypotension and cardiotoxicity
Snake C-type lectins (Snaclec)	NA	9 (±6)	6 (±8)	Platelet inhibition and activation via an array of receptors
Disintegrin	NA	6 (±5)	2 (±4)	Binding of integrins causing inhibition of platelet aggregation
